# Paralysie du nerf abducens droit révélant une pansinusite

**DOI:** 10.11604/pamj.2015.21.121.6978

**Published:** 2015-06-15

**Authors:** Adil Bouzidi, Said Iferkhass, Zine El Abidine Hansali, Mohammed Elmallaoui, Abdelkader Laktaoui

**Affiliations:** 1 Hôpital Militaire Moulay Ismail, Mekens, Maroc

**Keywords:** Paralysie du nerf abducens, fièvre, pansunisite, abducens nerve palsy, fever, pansunisitis

## Abstract

L'association entre la sinusite, en particulier, sphénoïdale et la paralysie oculomotrice a été déjà décrite dans la littérature, mais reste très rare. Nous rapportons un cas d'une patiente âgée de 14 ans sans antécédents pathologiques particuliers consultant pour une une paralysie du VI gauche survenant dans un contexte fébrile. L'examen ophtalmologiquet complété par un bilan radiologique, à révélé une pansunisite du même coté. Les auteures suggèrent que devant toute paralysie oculomotrice, et après avoir éliminé une étiologie tumorale, il faut rechercher à un foyer infectieux locorégional et le bien traite.

## Introduction

La paralysie de VI est fréquente et souvent acquise. L'association entre la sinusite en particulier sphénoïdale et la paralysie oculomotrice a été déjà décrite dans la littérature, mais reste très rare (HAIZAL). Nous rapportons un cas de paralysie du nerf abducens due à une pansinusite.

## Patient et observation

**Figure 1:**
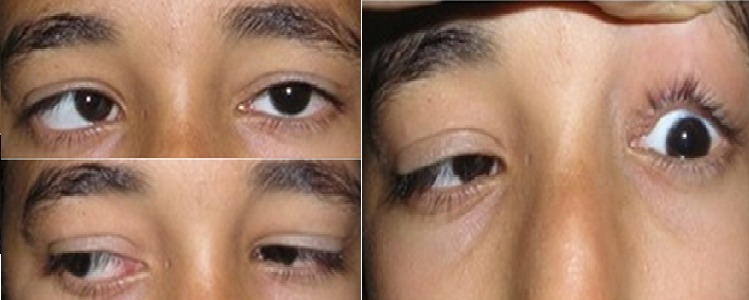
Limitation du muscle droit externe gauche avec hyperaction du muscle droit interne de l’œil droit

**Figure 2:**
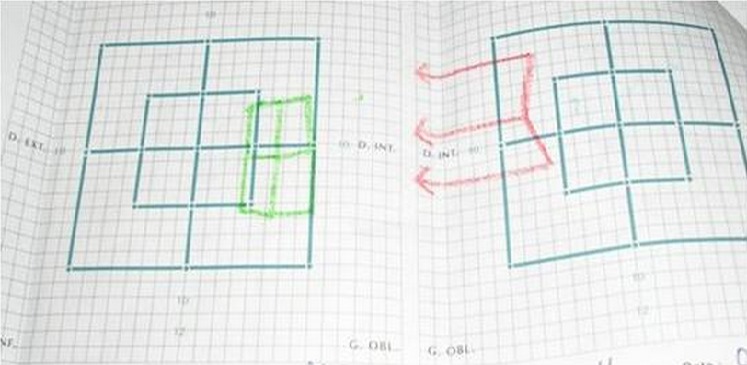
Lancaster: montre la paralysie du muscle droit externe gauche

**Figure 3:**
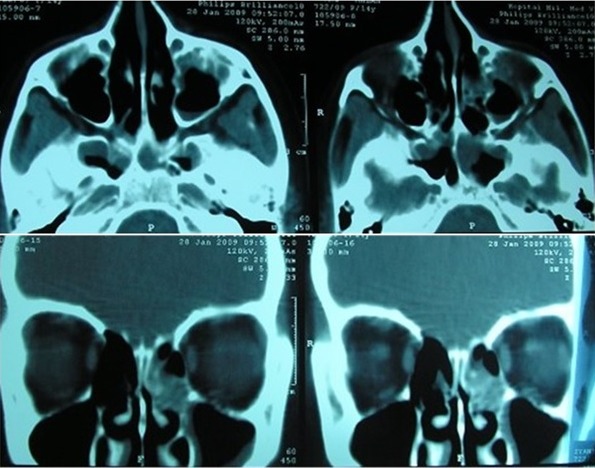
TDM orbitocérébral montrant une pansunisite

Patiente âgée de 14 ans sans antécédents pathologiques particuliers consulte pour un strabisme aigu convergent avec diplopie et céphalée chronique. Le tout évoluant dans un contexte subfébrile; l'examen oculomoteur montre une paralysie du VI gauche. L'examen ophtalmologique complet est complété par un bilan radiologique, un Lancaster et un avis neurologique et ORL. L'examen ophtalmologique montre une AV à 10/10 EN ODG avec un segment antérieur et postérieur normaux. L'examen oculomoteur objective une esotropie constante de l'OD de 30 dD (Figure 1) avec limitation du doit externe gauche et hyper action du droit interne OD en faveur d'une paralysie du VI de l'œil gauche confirmé par le Lancaster (Figure 2). L'examen neurologique et ORL est sans particularité. L'etudes de liquide céphalorachidien n'ont montré aucune preuve de la méningite. Le TDM orbitocérébrale (Figure 3) montre une sinusite maxilloethmoïdale et sphénoïdale surtout à gauche. En collaboration avec les confrères ORL, la patiente a été mise sous antibiothérapie par voie générale associée à une corticothérapie par voie orale pendant 7 jours débutée 48 heurs après. L’évolution était marquée deux mois après par la régression de la diplopie alors que les foyers sinusiens n'ont pas disparus totalement.

## Discussion

L'atteinte sphénoïde est une infection rare qui représente environ 3% de tous les cas de sinusite aiguë [1, 2]. Elle est généralement accompagnée par pansinusite; plus rarement, elle peut être latent et son diagnostic devient très difficile, surtout si sa première présentation est une complication [1, 2]; comme le cas de notre malade. Afin d’éviter des conséquences potentiellement mortelles, la reconnaissance précoce des déficits neurologiques focaux tels que les paralysies des nerfs crâniens et un transfert rapide à l'ORL est impératif [1]. Les symptômes de la sinusite peuvent être non spécifiques et variés. Les maux de tête, la congestion nasale, la fièvre et les troubles visuels sont des présentations les plus courantes, comme c'est le cas de notre patiente. Cependant, les formes isolées existent et rendent le diagnostic plus difficile. Dans ce cas, elles sont associées à une morbidité et une mortalité importantes [2]. L'atteinte Sphénoïdale est souvent mal diagnostiquée, puisque l'examen physique du sinus sphénoïde n'est pas toujours accessible, même avec l'endoscope flexible, et les patients ne sont pas tous symptomatiques [2]. Par ailleurs les sinus sphénoïdaux ne sont pas suffisamment visualisés avec les radiographies simples puisqu'elles passent à coté du diagnostique dans environ 26% des cas [3]. La Neuroimagerie (scanner ou IRM) est nécessaire pour diagnostiquer définitivement la sinusite sphénoïdale, en particulier dans les cas de sinusites réfractaires ou récurrentes [4]. Elle peut aider à confirmer le diagnostic et d'exclure les complications potentiellement mortelles [1, 5, 6].

L'atteinte du sixième nerf crânien varie selon l’âge. Chez les jeunes adultes, la paralysie du sixième nerf crânien est généralement idiopathique ou associée à une infection virale ou survient en cas d'une méningite [7]. Elle peut être liée à une irritation directe du nerf due à une augmentation de la pression intracrânienne. Cela peut se produire à la suite d'un traumatisme ou par effet de masse d'une lésion cérébrale [1]. Dans les infections virales du système nerveux central, la réaction immunitaire complexe ou l'infection elle-même, peuvent induire un déficit d'abduction similaire à celle observée en cas de paralysie du sixième nerf. Pour La plupart des auteurs, le risque de paralysie du nerf abducens est directement lié à la localisation anatomique de ce nerf par rapport aux sinus [2, 6]. En effet, le sphénoïde est adjacent à de nombreuses structures importantes telles que le sixième nerf crânien, la dure-mère, le sinus caverneux, et l'artère carotide interne [4, 8]. Les parois sphénoïdales peuvent être extrêmement mince, et parfois la cavité des sinus est séparée de ces structures adjacentes par seulement une mince barrière muqueuse, ce qui peut entraîner une irritation directe des, nerfs crâniens et des méninges, par propagation de l'inflammation et de l'infection en cas de sinusite [8]. Le rôle des stéroïdes dans le traitement de la sinusite est controversé. Elles peuvent réduire l'inflammation mais en même temps, diminuent la réponse immunitaire et exacerbent l'infection [1]. Nous rejoignons IM. HAIZUL et al [1] pour recommander l'introduction de la corticothérapie 48 heurs après le début de l'antibiothérapie. Chez les patients avec au moins trois épisodes documentés de la sinusite à l'année précédente, Chee et ses collègues [9] ont constaté qu'ils avaient une plus grande probabilité de dysfonctionnement immunitaire.

## Conclusion

Devant toute paralysie oculomotrice, et après avoir éliminé une étiologie tumorale, il faut rechercher à un foyer infectieux locorégional et le bien traiter.
